# Obesity and osteoarthritis in knee, hip and/or hand: An epidemiological study in the general population with 10 years follow-up

**DOI:** 10.1186/1471-2474-9-132

**Published:** 2008-10-02

**Authors:** Margreth Grotle, Kare B Hagen, Bard Natvig, Fredrik A Dahl, Tore K Kvien

**Affiliations:** 1 National resource centre for rehabilitation in rheumatology, Dept. of Rheumatology, Diakonhjemmet Hospital, Oslo, P.O.Box 23 Vinderen, 0319 Oslo, Norway; 2 FORMI, Division for neuroscience and musculoskeletal medicine, Ullevaal University Hospital, N-0407 Oslo, Norway; 3 Institute of general practice and community medicine, P.O.Box 1130 Blindern, University of Oslo, Norway; 4 Helse Sør-Øst Health Services Research Centre (HOKH), Akershus University Hospital, Mail drawer 95, NO-1474 Lørenskog, Norway; 5 Dept. of Rheumatology, Diakonhjemmet Hospital, Oslo, P.O.Box 23 Vinderen, 0319 Oslo, and Faculty of Medicine, P.O.Box 1130 Blindern, University of Oslo, Norway

## Abstract

**Background:**

Obesity is one of the most important risk factors for osteoarthritis (OA) in knee(s). However, the relationship between obesity and OA in hand(s) and hip(s) remains controversial and needs further investigation. The purpose of this study was to investigate the impact of obesity on incident osteoarthritis (OA) in hip, knee, and hand in a general population followed in 10 years.

**Methods:**

A total of 1854 people aged 24–76 years in 1994 participated in a Norwegian study on musculoskeletal pain in both 1994 and 2004. Participants with OA or rheumatoid arthritis in 1994 and those above 74 years in 1994 were excluded, leaving n = 1675 for the analyses. The main outcome measure was OA diagnosis at follow-up based on self-report. Obesity was defined by a body mass index (BMI) of 30 and above.

**Results:**

At 10-years follow-up the incidence rates were 5.8% (CI 4.3–7.3) for hip OA, 7.3% (CI 5.7–9.0) for knee OA, and 5.6% (CI 4.2–7.1) for hand OA. When adjusting for age, gender, work status and leisure time activities, a high BMI (> 30) was significantly associated with knee OA (OR 2.81; 95%CI 1.32–5.96), and a dose-response relationship was found for this association. Obesity was also significantly associated with hand OA (OR 2.59; 1.08–6.19), but not with hip OA (OR 1.11; 0.41–2.97). There was no statistically significant interaction effect between BMI and gender, age or any of the other confounding variables.

**Conclusion:**

A high BMI was significantly associated with knee OA and hand OA, but not with hip OA.

## Background

Obesity is considered to be one of the most important risk factors for osteoarthritis (OA) in knee(s). Numerous longitudinal studies show a strong association between obesity, defined as a body mass index (BMI) above 30, and radiographic knee OA, e.g. in the Framingham Study [1], the Chingford Study [2], the Baltimore Longitudinal Study of Aging [3], the John Hopkins Precursors Study [4], and in longitudinal studies in UK [5] and the Netherlands [6]. Thus, the WHO initiative on counteracting obesity also accepts OA as a consequence of obesity [7].

However, the relationship between obesity and OA in hand(s) and hip(s) remains controversial [7,8]. A significant relationship between obesity and radiographic hip OA has been found in some cross-sectional studies [9-13] as well as in longitudinal studies [14-16]. In large longitudinal studies of Gelber et al [4] and Reijman et al [6] high BMI was not associated with hip OA. Large cross-sectional studies have failed to show a significant association between obesity and hand OA in either males or females [17-19], whereas some prospective data have demonstrated that obesity predicted hand OA [14,20].

Thus, there is a need for further exploration of the influence of obesity on OA, in particular related to hip OA and hand OA: The aim of this study was to investigate the longer term impact of obesity for OA in hip, knee, and/or hand in a general population followed in 10 years. The main hypothesis was that high BMI is a significant risk factor for OA in the weight bearing joints (hips and knees), whereas we expected no significant association with hand OA.

## Methods

### Study sample and setting

This is a prospective cohort study on musculoskeletal pain in Ullensaker [21,22]. Ullensaker is a municipality 40 kilometres northeast of Oslo with 23.500 inhabitants, many of them being commuters to Oslo, the capital of Norway.

In 1994, all the 4589 inhabitants born in 1918–20, 1928–30, 1938–40, 1948–50, 1958–60 and 1968–70 were sent a postal questionnaire about musculoskeletal pain: 63% (n = 2891) responded to this survey. The responders in 1994 were more often women and in the middle aged groups. Of these, 64% (n = 1854) responded to the 2004-follow-up survey 10 years later. We excluded people who reported OA in any joint or rheumatoid arthritis in 1994 (n = 134) and people born in 1918–1920 (n = 45) due to low number and response. A second mailing of the questionnaire to non-responders was sent after six weeks. The number of individuals available for the current analyses was 1675.

The Regional Committee for Medical Research Ethics and The Norwegian Data Inspectorate approved the study.

### Report of osteoarthritis in hip, knee and hand

The presence of OA in hip, knee, and/or hand was obtained by the item "Have you ever been diagnosed with osteoarthritis in hip/knee/hand by a MD and/or x-ray?" Respondents could mark for yes in hip, knee and/or hand. There was no explicit alternative for no, and when a subject did not report yes, the response was defaulted to no. This has the benefit of reducing the effort required to complete the form, but also has the effect that we cannot distinguish between a no and a missing value.

### Risk factors

Body mass index (BMI) (weight/height^2^) was calculated based on the self-reported body weight and height, and entered as a categorical variable (classified as BMI < 20, 20–25, 26–30, and > 30). Obesity was defined by a body mass index (BMI) of 30 and above. Age, gender, work status, and leisure physical activity were included in the analyses as potential confounding factors. Age was grouped according to the age cohorts (from 1928–30, 1938–40 etc until 1968–70). Work status was recorded as employed, homemakers, non-employed, disability pensioned, age pensioned, and student. Frequency of leisure physical activity was recorded as none, < 2 hours per week, 2–4 hours per week, and > 4 hours per week.

### Data analysis and statistical methods

The incidence of OA in hip, knee, and hand during the 10-year follow-up period was calculated with 95% confidence intervals (CI). Distribution of the dependent and indendent variables were analysed by frequency analyses. Odds ratios (OR) were estimated in multivariate logistic regression analyses. We defined a BMI of 20–25 as the reference category since this represents a normal BMI. The odds ratio can be interpreted as an approximate relative risk when the events are rare (< 10%). First, the association between baseline BMI and the outcome variables were analysed adjusting for age and gender. Second, the analyses were also adjusted for the other possible risk factors (age, gender, work status, and leisure physical activity). Finally, the association was tested for interaction between BMI and the other independent variables. Exposure variables that remained significant at the 5% level in the multivariate model were considered to have an independent association with the risk of OA. Analyses were performed using the SPSS software, version 14.0 (SPSS Inc., Chicago, USA).

## Results

The 1675 respondents (943 women), initially free of OA or rheumatoid arthritis in 1994, had a mean (SD) baseline age of 41.8 (12.9) years (range 24 to 66 years), and 76.6% were employed. Mean BMI in 1994 was 24.2 (3.3) (median 23.9), with 5.3% having a BMI above 30. In 2004 the mean BMI was 25.6 (3.9) (median 25.2) with 12.1% having a BMI above 30. There was no difference in the BMI distribution between responders and non-responders in 2004 (p = 0.909).

From 1994 to 2004, 5.8% (CI 4.3–7.3) developed OA in the hips, 7.3% (CI 5.7–9.0) in the knees, and 5.6% (CI 4.2–7.1) in the hands. The 10-year incidence for hand OA was significantly (p = 0.001) higher among women (5.6%, CI 4.2–7.1) compared to men (2.5%, CI 1.3–3.6), whereas no significant differences between the genders were found in the incidence of OA in hip (5.8%, CI 4.3–7.3 for women and 3.8%, CI 2.4–5.2 for men, p = 0.060) and knee (7.3%, CI 5.7–9.0 for women and 6.2%, CI 4.4–7.9 for men, p = 0.346) (Table 1). Only a minor proportion of the responders reported OA in more than one body region: 1.3% reported hand/knee OA, 1.4% hip/knee OA, 1.1% hip/hand OA, and 0.6% reported OA in all three body regions (hip, knee, and hand).

**Table 1:** 10-years incidence of osteoarthritis in hip, knee, and hand (in 2004).

All (n = 1675)	Hip (N = 83)	Knee OA (N = 114)	Hand OA (N = 71)
BMI			
< 20 (n = 133)	3 (2.3)	7 (5.3)	8 (6.0)
20 – 25 (n = 918)	47(5.1)	45 (4.9)	36 (3.9)
26–30 (n = 504)	28 (5.6)	49 (9.7)	20(4.0)
> 30 (n = 87)	5 (5.7)	11 (12.6)	7 (8.0)
Missing cases (n = 33)	-	-	-
Gender			
Male (n = 732)	28 (3.8)	45 (6.1)	18 (2.5)
Female (n = 943)	55 (5.8)	69 (6.8)	53 (5.6)
Age			
1928–1930 (n = 158)	22 (13.9)	26 (16.5)	11 (15.5)
1938–1940 (n = 312)	31 (9.9)	37 (11.9)	22 (7.1)
1948–1950 (n = 453)	23 (5.1)	33 (7.3)	27 (6.0)
1958–1960 (n = 339)	6 (1.8)	10 (2.9)	10 (2.9)
1968–1970 (n = 413)	1 (0.2)	8 (1.9)	1 (1.4)
Work status			
Employed (n = 1277)	51 (4.0)	74 (5.8)	46 (3.6)
Homeworkers (n = 117)	10 (8.5)	11 (9.4)	10 (8.5)
Non-employed (n = 53)	2 (3.8)	4 (7.5)	1 (1.9)
Disability pensioned (n = 86)	12 (14.0)	16 (18.6)	11 (12.8)
Age pensioned (n = 55)	5 (9.1)	7 (12.7)	3 (5.5)
Student (n = 76)	1 (1.3)	0	1 (1.3)
Missing cases (n = 11)	-	-	-
Leisure time physical activities			
< 2 hours/week (n = 810)	48 (5.9)	55 (6.8)	32 (4.0)
2–4 hours/week (n = 606)	36 (5.8)	41 (6.8)	23 (3.8)
> 4 hours/week (n = 228)	6 (2.6)	12 (5.3)	12 (5.3)
Missing cases (n = 31)	-	-	-

The results are presented according to body mass index, gender, age, work status, and level of leisure time activities (in 1994) in the general population of Ullensaker, Norway, initially free of rheumatic diseases (OA and RA) in 1994 (number and percentages of rows in parantheses).

Table 1 shows that there were few cases with OA in the lowest (< 20) and highest (> 30) categories of BMI. Obesity, defined as BMI above 30, was significantly associated with knee OA 10 years later with an OR of 2.8, and a dose-response relationship was found for this association (Table 2). Table 2 also shows that obesity was significantly associated with hand OA with an OR of 2.6 in the multivariate analysis. The analyses did not reveal any indication of an association between obesity and hip OA. For hand- and knee OA, there was no statistically significant interaction effect between gender and BMI, age and BMI, or any of the other confounding variables and BMI.

**Table 2:** Association between body mass index and osteoarthritis in hip, knee, and hand in the general population.

	Hip OA N = 83		Knee OA N = 114		Hand OA N = 71	
	
	Adjusted for age and gender	Multivariate-adjusted^1^	Adjusted for age and gender	Multivariate-adjusted^1^	Adjusted for age and gender	Multivariate-adjusted^1^
Body Mass Index^3^						
< 20	0.53 (0.16–1.78)	0.51 (0.15–1.72)	1.30 (0.56–3.02)	1.33 (0.57–3.10)	1.70 (0.75–3.85)	1.71 (0.75–3.89)
20–25	1	1	1	1	1	1
26–30	0.94 (0.57–1.55)	0.82 (0.48–1.39)	1.91 (1.24–2.96)	2.02 (1.29–3.16)	0.98 (0.55–1.74)	0.98 (0.54–1.78)
> 30	1.07 (0.40–2.84)	1.11 (0.41–2.97)	2.77 (1.35–5.71)	2.81 (1.32–5.96)	2.24 (0.95–5.33)	2.59 (1.08–6.19)

Results are presented by odds ratios (OR) with 95% confidence intervals (CI) adjusted for age and gender (left column) and multivariate-adjusted OR (right column).

^1^ Adjusted for age, gender, work type, leisure time activities, ^2 ^continuous variable, ^3 ^We defined a BMI of 20–25 as the reference category since this represents a normal BMI.

Due to missing data for 33 cases on BMI the total number in the present analyses is 1642.

## Discussion

In this population-based study of people without OA or rheumatoid arthritis at baseline and followed for 10 years we found that BMI was a consistent and dose-related predictor of knee OA in all types of analyses. We also found that obesity (BMI > 30) was a significant independent predictor of incident hand OA. We did not find any indication of a relationship between BMI and occurrence of hip OA.

The main limitation of this study is that the epidemiological case definition of clinically diagnosed OA was based on self-report through the response to a written question and not based on radiographic evidence. However, the present outcome question referred to OA diagnosed by a medical doctor, and not only to pain in the actual body regions. In a previous study, we found that this OA question to a large extent differentiated between OA and musculoskeletal pain in the actual joints as approximately 18% of those who reported pain also reported OA in the same joint [23]. Moreover, the present 10-year incidence estimates of hip and knee OA is at the same level as the radiographic based estimates in the recent published study of Reijman et al [4,6], who found that 5.5 % and 3.9% developed radiographic incident knee OA and hip OA, respectively, during a mean follow-up time of 6.6 years. However, it's obvious that with the current definition this survey will not capture OA without pain or other symptoms. Hence, the current findings relate to obesity and symptomatic OA. It can be argued that the relationship between obesity and OA is mostly of interest in symptomatic OA, and that radiographically diagnosed ("silent") OA perhaps has less practical interest for clinicians. In a research perspective there is definitely a need to explore the precision of self-reported diagnosis of OA.

Another limitation may be a response of 64% with the lowest response among males and among the youngest and oldest age groups. The oldest age group born in 1918–1920 was excluded. As expected, a low incidence of OA was observed in the youngest age group. Thus the results in the present study are not likely to be influenced by low response in this group. However, there was a higher proportion of women than men in this sample, which should be taken into account when interpreting these results. Furthermore, the number of OA cases was small among subjects with BMI < 20 and > 30, and the results for these groups should be interpreted with caution. A final limitation is that we lack data on the reliability of self-reported BMI. However, self-reported and measured BMI mostly correlate well, even though there has been reported a tendency to under report the BMI, especially among adolescents with overweight [24].

The main strength of this study is that it was carried out in the general population with people aged between 24 and 66 years at the start of the study. Furthermore, this prospective study had a relatively large number of respondents taking into consideration the long follow-up period of 10 years. Population-based studies are important as the cases are unselected for severity in comparison with hospital-based populations.

The present results confirm that obesity is a strong determinant for knee OA [1-6]. A non-significant relationship between obesity and hip OA is also in line with two previous large longitudinal studies [4,6]. However, some other large prospective studies have reported different results regarding the impact of high BMI on hip OA [14-16]. A systematic review of the influence of obesity on hip OA included five longitudinal and seven cross-sectional studies, and found moderate evidence for a positive association between obesity and hip OA with an OR of approximately 2 [25].

Similarly, it is an open question whether obesity is associated with an increased risk of hand OA [7]. Our results support that a relation is possible, but the results were less consistent and overall weaker than the association that were observed between BMI and knee OA. Furthermore, we did not find any dose-response effect so this finding should be interpreted with carefulness. Since only a minor proportion of 1.3% reported OA in both hand and knee, it's not likely that this overlap can explain the BMI and hand OA association.

Most studies up to now on the association between obesity and hand OA have been cross-sectional [17-19], but there has been some prospective data showing that obesity predicted hand OA [14,20]. Both mechanical and systemic mechanisms have been put forth to explain the effect of obesity on hand OA, but the reason for this association is currently unknown [24]. Our finding may support that OA has an important systemic component, and not only a mechanical loading component.

## Conclusion

This study supports that obesity is an independent weak risk factor for hand OA. None of the analyses give any indication of an association between BMI and hip OA. On the other side, this study also confirms that obesity is an important risk factor for development of knee OA. The future research agenda should focus on how community action programmes focusing on obesity may impact occurrence of OA (primary and secondary prevention) and symptom improvement in patients with existing OA (tertiary prevention).

## Competing interests

The authors declare that they have no competing interests.

## Authors' contributions

KBH, TKK and BN developed the study design. MG carried out the statistical analyses supervised by FAD, and all five authors have taken active part in the analytic approach and writing of the manuscript. All authors have read and approved the final manuscript.

## Pre-publication history

The pre-publication history for this paper can be accessed here:


